# Mood Lifters: A Peer-Led Mental Health Program for Older Adults via Video Conferencing

**DOI:** 10.1093/geroni/igab046.3137

**Published:** 2021-12-17

**Authors:** Courtney Funk, Rebecca Ferber, Anne Harrington, Jennifer Porte, Cathleen Connell, Susan Maixner, Scott Roberts

**Affiliations:** 1 University of Michigan School of Public Health, Ann Arbor, Michigan, United States; 2 University of Michigan, Ann Arbor, Michigan, United States; 3 The University of Michigan, ANN ARBOR, Michigan, United States; 4 Mood Lifters, LLC, Ann Arbor, Michigan, United States

## Abstract

Effective and scalable mental health programs are greatly needed for older adults, given the vast majority in need do not receive formal mental health services. In this study, we adapted Mood Lifters—a peer-led, community-based program promoting mental well-being—to address the unique needs of older adults. The 14 weekly program sessions were delivered via Zoom. Twelve older adults (mean age = 69.7 years; 4 men, 8 women) enrolled; 9 completed the program (2 of 3 dropouts were due to health issues). A battery of validated measures administered within one week before and after the program assessed domains including depression and anxiety, stress management, and health behaviors. Compared to baseline, participants who completed the program showed significant improvements in perceived stress (p=0.03), sleep quality (p=0.02), and emotion regulation via cognitive reappraisal (p=0.06). Depression and anxiety symptoms (assessed by the Geriatric Depression and Anxiety Scales, respectively) were lower at program completion, although improvements were not statistically significant. No significant changes from pre- to post-test were reported in loneliness and health behaviors. Participant ratings of program satisfaction were very high (mean = 4.78/5, with 1=poor, 5=excellent). Results from this pilot test of Mood Lifters for Seniors suggest it is feasible and acceptable for outreach to older adults, with preliminary evidence of benefits in several domains related to mental health and wellness. Future randomized trials with larger, more diverse samples will be necessary to confirm program benefits.

